# Number of intraepithelial lymphocytes and presence of a subepithelial band in normal colonic mucosa differs according to stainings and evaluation method

**DOI:** 10.1016/j.jpi.2024.100374

**Published:** 2024-03-24

**Authors:** Anne-Marie Kanstrup Fiehn, Peter Johan Heiberg Engel, Ulla Engel, Dea Natalie Munch Jepsen, Thomas Blixt, Julie Rasmussen, Signe Wildt, Wojciech Cebula, Andreea-Raluca Diac, Lars Kristian Munck

**Affiliations:** a Department of Pathology, Zealand University Hospital Roskilde, Sygehusvej 9, 4000 Roskilde, Denmark; b Department of Clinical Medicine, University of Copenhagen, Blegdamsvej 3B, 2200 Copenhagen, Denmark; c Department of Pathology, Copenhagen University Hospital Hvidovre, Kettegård Allé 30, 2650 Hvidovre, Denmark; d Center for Surgical Science, Zealand University Hospital Køge, Lykkebækvej 1, 4600 Køge, Denmark; e Department of Medical Gastroenterology, Zealand University Hospital Køge, Lykkebækvej 1, 4600 Køge, Denmark; f GastroUnit, Department of Medical Gastroenterology, Copenhagen University Hospital Hvidovre, Kettegård Allé 30, 2650 Hvidovre, Denmark; g Department of Medical Gastroenterology, Zealand University Hospital Nykøbing Falster, Fjordvej 15, 4800 Nykøbing Falster, Denmark

**Keywords:** Normal histology, Histopathology, Intestinal mucosa, Microscopic colitis, Immunohistochemical staining, Digital pathology

## Abstract

Chronic watery diarrhea is a frequent symptom. In approximately 10% of the patients, a diagnosis of microscopic colitis (MC) is established. The diagnosis relies on specific, but sometimes subtle, histopathological findings. As the histology of normal intestinal mucosa vary, discriminating subtle features of MC from normal tissue can be challenging and therefore auxiliary stainings are increasingly used. The aim of this study was to determine the variance in number of intraepithelial lymphocytes (IELs) and presence of a subepithelial band in normal ileum and colonic mucosa, according to different stains and digital assessment. Sixty-one patients without diarrhea referred to screening colonoscopy due to a positive feacal blood test and presenting with endoscopically normal mucosa were included. Basic histological features, number of IELs, and thickness of a subepithelial band was manually evaluated and a deep learning-based algorithm was developed to digitally determine the number of IELs in each of the two compartments; surface epithelium and cryptal epithelium, and the density of lymphocytes in the lamina propria compartment. The number of IELs was significantly higher on CD3-stained slides compared with slides stained with Hematoxylin-and-Eosin (HE) (*p*<0.001), and even higher numbers were reached using digital analysis. No significant difference between right and left colon in IELs or density of CD3-positive lymphocytes in lamina propria was found. No subepithelial band was present in HE-stained slides while a thin band was visualized on special stains. Conclusively, in this cohort of prospectively collected ileum and colonic biopsies from asymptomatic patients, the range of IELs and detection of a subepithelial collagenous band varied depending on the stain and method used for assessment. As assessment of biopsies from patients with diarrhea constitute a considerable workload in the pathology departments digital image analysis is highly desired. Knowledge provided by the present study highlight important differences that should be considered before introducing this method in the clinic.

## Highlights

• Histology of intestinal mucosa in asymptomatic individuals varies widely.
• Number of intraepithelial lymphocytes (IELs) depends on stain.
• An AI-based algorithm for lymphocyte counts was developed.
• No significant difference in number of IELs between right and left colon was seen.
• Identification of a subepithelial band depends on the used staining.

## Introduction

In approximately 10% of patients with chronic watery diarrhea, a diagnosis of microscopic colitis (MC) is established.^1^, ^2^, ^3^, ^4^ The incidence varies up to 25 per 100,000 inhabitants and have exceeded the more well-known entity inflammatory bowel disease in high incidence areas.^5^^,^^6^ MC has a severely negative impact on quality of life.^7^ In patients with MC, endoscopy show a macroscopically normal or near-normal intestinal mucosa, while the histopathological examination reveals more or less specific morphological features.^8^ As variation in the normal histology of intestinal mucosa exists, it can be challenging to differentiate normal tissue from MC cases with less pronounced changes. In borderline cases, additional stainings, beyond the basic Hematoxylin-and-Eosin (HE) can be helpful and are widely used by pathologists everywhere in Europe.^9^

The two major subgroups of MC are collagenous colitis (CC) and lymphocytic colitis (LC). The histological criteria are based on HE-stained tissue sections.^10^^,^^11^ In addition to increased infiltration of lymphoplasmacytic cells in the lamina propria, an increased number of intraepithelial lymphocytes (IELs) in the surface epithelium exceeding 20 per 100 epithelial cells are required for a diagnosis of LC. A diagnosis of CC requires presence of a subepithelial collagen band exceeding 10 μm in thickness. Incomplete MC (MCi) is considered a separate diagnostic entity defined as patients with clinical signs of MC, but where the histological criteria for LC and CC are not completely met.^8^^,^^10^ For the incomplete forms of MC, between 10 and 20 IELs are suggested in order to consider a diagnosis of LCi, and a thickness of the collagen band between 5 and 10 μm for CCi. The incidence of MC varies widely between otherwise comparable countries and centers.^12^ Part of this difference might rely on variations in staining methods, as use of supplementary stains increase the sensitivity, but at the same time, represent a risk of overdiagnosis.^13^

In contrary, several previous studies that have examined the histology of intestinal mucosa in healthy individuals have reported that a few lymphocytes are present in the surface epithelium of the normal colon mucosa but usually reported to be <5 per 100 epithelial cells.^14^, ^15^, ^16^ In ileum, epithelium numbers reported varies from <5 to 13.^16^, ^17^, ^18^, ^19^ The number of inflammatory cells in the surface epithelium and in lamina propria is described to be higher in the right side of the colon decreasing towards rectum.^20^^,^^21^ A thin basement membrane with a thickness of up to 3 μm is described to be located beneath the surface epithelium^15^^,^^22^ but no thickened subepithelial collagenous band should be present.^23^

The aim of this study was to describe the variance in the number of IELs and possible presence of a subepithelial collagenous band in biopsies from macroscopically normal ileum and colon mucosa using a variety of stainings in asymptomatic patients. Furthermore, an artificial intelligence (AI)-based algorithm was developed for digital counting of lymphocytes in different compartments of the colon mucosa. Knowledge of differences in number of IELs and in presence of a collagenous band obtained as a result merely of different stainings is fundamental to the pathologist in order to avoid overdiagnostics. In the same way, differences produced by AI is important to reveal and be aware of before considering the introducing automated image analysis in the clinic.

## Materials and methods

### Patients

This is a prospective study including a cohort of patients with an ileo-colonoscopy performed at two endoscopy sites (Zealand University Hospital, Køge, Denmark and Nykøbing Falster Hospital, Denmark), both located in Region Zealand in the southern part of Denmark and having a population of more than 800,000 inhabitants. The study cohort consisted of patients referred for a colonoscopy due to a positive fecal blood test as part of the national colorectal cancer screening programme. No patients with clinical symptoms were included. Written study information was sent to the patient along with information of the procedure. Only patients ≥18 years, without prior history of chronic inflammatory bowel disease or MC, were invited to participate. Exclusion criteria were; reported diarrhea at the time of endoscopy (defined as >3 daily stools or ≥1 watery daily stool as a mean of 1 week before colonic cleansing), endoscopy with macroscopic inflammation of the colon mucosa, or a histological examination revealing extensive active inflammation, high-grade neoplasia or cancer. A diagnosis of adenoma with low-grade neoplasia was allowed if the biopsies were taken at a distance of at least 2 cm from the adenoma. From each patient, the endoscopist attempted to obtain two biopsies from the ileum, the right side of colon (coecum or ascending colon), and the left side of colon (descending or sigmoid colon). Biopsies from the three localizations were submitted in separate containers. Clinical information including sex, age, macroscopic changes, number of daily stools, and stool consistency was registered. Written informed consent was obtained, and patients were enrolled prospectively from October 2019 to February 2020.

### Staining and scanning of slides

Sections with a thickness of 4 μm were cut from the formalin-fixed paraffin-embedded tissue blocks at the Department of Pathology, Zealand University Hospital, Roskilde, Denmark and all cases were stained with HE, Van Gieson (VG), Masson Trichrome (MT), and Weigert-Alcian-Sirius (WAS), according to instructions provided by the manufacturer. The stains were processed using Tissue-Tek Prisma Automated Slide Stainer (Sakura, Denmark). The immunohistochemical (IHC) staining was performed using anti-human CD3 clone LN10 (Novo Castra, United Kingdom) on the Omnis autostainer platform (Dako, Denmark). Slides were deparaffinized, and antigen retrieval was performed by immersing slides in EnVision™ FLEX Target Retrieval Solution, High pH (Dako, Denmark) and heating to 97°C for 20 min followed by incubation with the primary CD3 antibody (dilution 1:50) for 30 min. Reactions were detected using EnVision™ FLEX+/HRP Detection Reagent and visualized with Envision DAB+ substrate according to instructions provided by the manufacturer. Slides were counterstained with hematoxylin.

All slides were digitized at 20× magnification using a Leica SCN400 slide scanner (Leica Biosystems, Nussloch Germany). Subsequently, CD3-stained digitized images were uploaded and processed using the Visiopharm Quantitative Digital Pathology software (Hoersholm, Denmark), version 2023.01.

### Manual histopathological evaluation

All slides were blinded and evaluated by two independent gastrointestinal (GI) pathologists with more than 20 years of experience. For all three locations, the following parameters were evaluated based on HE-stained slides according to the Geboes scale^24^; architecture (no, mild, moderate, or severe abnormalities), chronic inflammatory infiltrate (no, mild, moderate, or marked increase), eosinophils in lamina propria (no, mild, moderate, or marked increase), neutrophils in lamina propria (no, mild, moderate, or marked increase), neutrophils in epithelium (none, <5% of crypts, 5–50% of crypts, or >50% of crypts involved), crypt destruction (none, discrete and locally, discrete but diffuse, or unequivocal), and erosion/ulceration (none, regenerating epithelium, probable erosion, unequivocal erosion, or ulceration/granulation tissue). The number of IELs in the surface epithelium was assessed as number of positive lymphocytes per 100 epithelial cells as continuous numbers by both pathologists independently, first by a HE and next by CD3 stain. Presence of a subepithelial band was evaluated using HE-, VG-, MT-, and WAS-stained slides, and was assessed as continuous numbers by one pathologist. The number of IELs and a possible subepithelial band was assessed in hot spots (not overlying lymphocyte aggregates in the lamina propria) and only in areas with optimal orientation of the biopsies with the crypts cut perpendicular to the surface.

### Digital evaluation

**Fig. 1:**
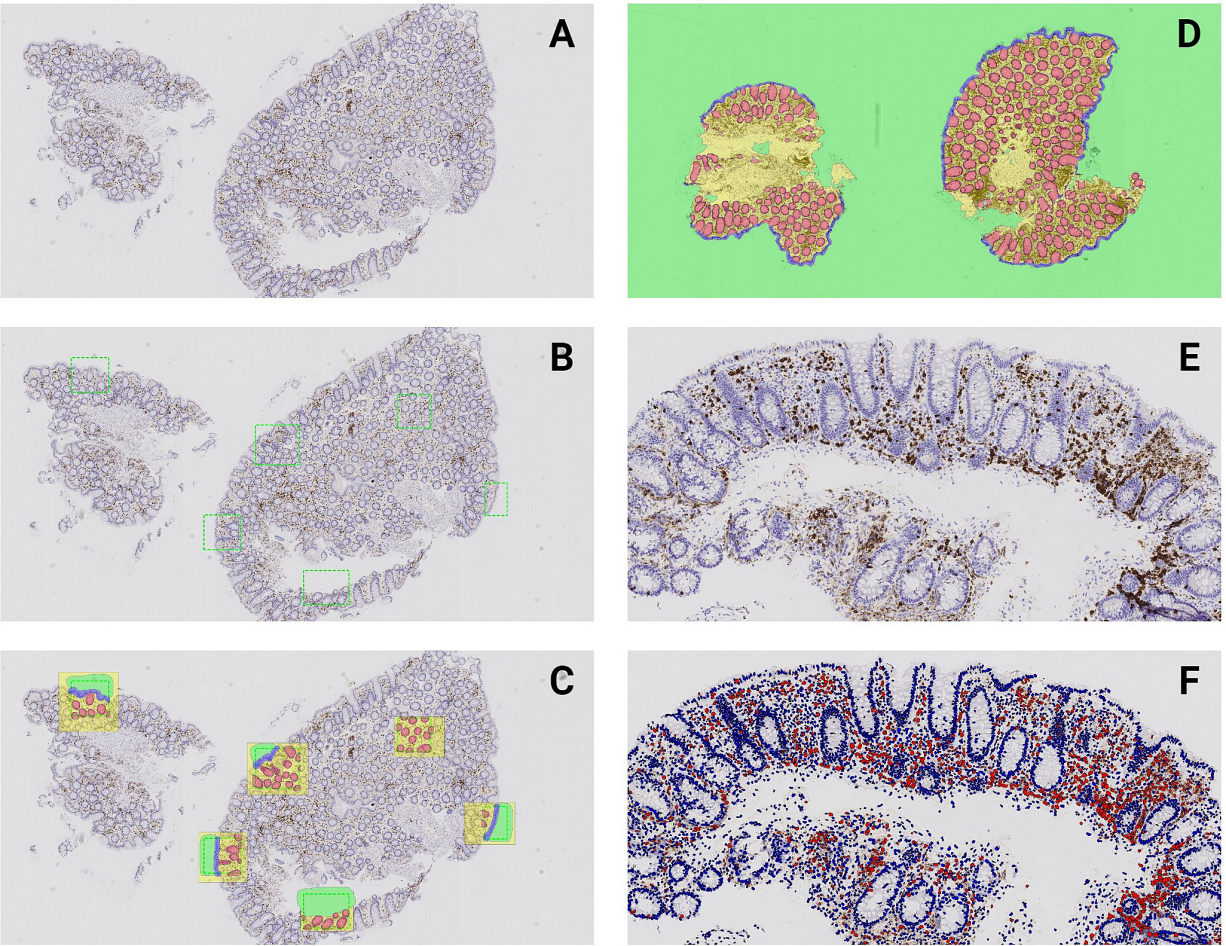
The process of developing two deep learning-based algorithms. A–D illustrates the segmentation algorithm (magnification 2.5×) and E–F the counting algorithm (magnification 10×). (A) Section stained with CD3; (B) regions of interest (ROIs) are drawn on the slide; (C) all structures in the ROIs are labeled as background (green), surface epithelium (blue), crypt epithelium (red), or lamina propria (yellow); (D) following training the performance of the algorithm was assessed in an independent cohort; (E) a CD3-stained slide from the study cohort before processing; (F) CD3-positive nuclei are visualized in red color and negative nuclei in blue color (Number of CD3-positive cells in surface epithelium, and crypt epithelium, and densities in lamina propria are reported for each compartment separately. The segmentation into the three compartments are not shown on the image.).

A deep learning-based application protocol package (APP) was developed to perform digital counts of CD3-positive lymphocytes in the colon mucosa with separate counts being obtained for three individual compartments; surface epithelium, crypt epithelium, and lamina propria (including all tissue not classified as epithelium). To obtain this, two APPs were developed and executed sequentially. First, an APP segmenting the tissue into four morphological classes (surface epithelium, crypt epithelium, lamina propria, and background) was developed using a deep learning classifier (DeepLabv3+). An independent training set consisting of 12 slides with biopsies diagnosed as normal colon mucosa and stained with CD3 was used as basis for developing the APP. The selected training set consisted of slides representing a spectrum of normal colon mucosa with variations in IHC staining intensity and morphology. A GI pathologist manually selected representative regions of interest (ROIs) and labeled all of the tissue structures in these ROIs into one of the four aforementioned classes representing the basis defining the ground-truth data. The training was conducted at 5× magnification. A loss function of the Visiopharm AI Author module shown during the learning process of the convolution neural network (CNN) was followed and training was deemed satisfactory at 50,000 iterations. Following training, the APP was executed across whole-slide images of the complete training set consisting of 12 slides and an additional independent validation set consisting of 22 slides also originating from patients diagnosed with normal colon mucosa. Neither the training set nor the validation set was part of the study cohort. The result was evaluated manually by a pathologist. Areas with unsatisfying segmentation were identified and additional ROIs with training labels were provided. The APP was retrained until segmentation of the images into the four morphological classes were comparable to the manual evaluation by the pathologist. Next, separate lymphocyte counts were performed in each of the three compartments; surface epithelium, crypt epithelium, and lamina propria. For the lymphocyte quantification, a pre-trained AI APP (Nuclei detection, AI Brightfield, Visiopharm) was used as basis and adapted as necessary. The APP had initially been trained at 20× magnification leveraging a deep learning classifier (U-Net, 200,000 iterations) to identify background, nuclei, and nuclear boundary and is available as a ready-to-use APP without additional training (https://visiopharm.com/app-center/app/nuclei-detection-ai-brightfield). Discriminating the CD3-positive from CD3-negative nuclei were performed by customizing the post-processing steps and setting a threshold based on the IHC staining intensity of the present cohort. IELs in each of the two separate compartments, surface epithelium and crypt epithelium, were counted as number of positive lymphocytes per 100 epithelial cells. In lamina propria, the density was calculated as the number of CD3-positive cells/area in mm^2^. Fig. 1 illustrates the process for obtaining the digital data.

### Statistics

Manual and digital IEL counts are presented as means with 95% confidence intervals (CIs) and ranges, and visualized with box plots. For each pathologist, manual lymphocyte counts obtained by HE was compared to counts by CD3-stained slides. The manual CD3 lymphocyte counts obtained by the pathologists were compared to the digital lymphocyte counts in the surface epithelium. The digital lymphocyte counts in each compartment (surface epithelium, crypt epithelium, and lamina propria) were compared according to right and left side of the colon. For the abovementioned statistical comparisons, a paired *t*-test was used, and a *p*-value of <0.05 was considered statistically significant. Data on thickness of the collagenous band are presented as frequencies with percentages according to different staining methods. Pairwise comparisons of the thickness of the collagenous band according to different stainings were performed using the exact Wilcoxon-Pratt signed-rank test. To adjust for multiple comparisons, the Bonferroni correction was used, and by dividing 0.05 with the number of comparisons performed at each intestinal location (0.05/6) a *p*-value of <0.008 was considered statistically significant. For statistical analyses and creation of box plots, the statistical software R, version 4.3.1, was used (R Core Team (2023). R: A language and environment for statistical computing. R Foundation for Statistical Computing, Vienna, Austria. URL https://www.R-project.org/.), including packages LMMstar,^25^ dplyr,^26^ ggplot2,^27^ and coin package.^28^

### Ethics

The study was approved by the Committee on Health Research Ethics of Regions Zealand, Denmark (SJ-631), and registered by the Danish Data Protection Agency (REG-113-2017).

## Results

### Patients

In total, 78 patients were invited and accepted to participate in the study. Sixteen patients were excluded, due to diarrhea during the week prior to colonic cleansing, and one patient due to adenocarcinoma in an adenoma. Thus, 61 patients were included in the study. For all patients, at least two biopsies were available from the right and the left side of colon. Biopsies from the ileum were obtained from 52 patients. Age, sex, number of daily stools, and macroscopic appearance of the mucosa are shown in Table 1. After a minimum of 42 month following the colonoscopy, the national pathology database which contains complete histological data for the total cohort was checked. No patients were diagnosed with histological inflammation in biopsies from the GI tract during this period. No clinical follow-up was available.

**Table 1:** Clinical characteristics of the included patients.

Sex, *n*	
- Male	35
- Female	26
Age, mean (range)	
- Male	62.97 (50–74)
- Female	62.58 (50–74)
Number of daily stools	
- 0–1	37
- 2–3	24
Number of daily watery stools	
- 0	61
Macroscopic appearance, *n*	
- Normal	25
- Adenoma	36

### Histopathological parameters

A detailed description of the registered parameters is shown in Table 2. None of the two pathologists reported architectural changes, crypt degeneration, or erosions/ulcers in the ileal mucosa. One pathologist registered a mild chronic inflammatory infiltrate, and an increased number of eosinophils in a few patients, and in one patient neutrophils and cryptitis occurred rarely. The other pathologist did not register any abnormalities.

**Table 2:** Histological characteristics in terminal ileum, right, and left colon mucosa, assessed manually by the two pathologists on Hematoxylin-and-Eosin-stained slides.

	Architectural disturbance	Chronic inflammation in lamina propria	Eosinophils in lamina propria	Neutrophils in lamina propria	Cryptitis	Crypt degeneration	Erosion/ulceration
Ileum, pathologist 1 (*n*=51)	None: 51	None: 46	None: 43	None: 50	None: 50	None: 51	None: 51
		Mild: 5	Mild: 4	Mild: 1	<5%: 1		
			Moderate: 3				
			Marked: 1				
Ileum, pathologist 2 (*n*=51)	None: 51	None: 51	None: 51	None: 51	None: 51	None: 51	None: 51
Right colon, pathologist 1 (*n*=61)	None: 60	None: 30	None: 35	None: 61	None: 59	None: 61	None: 61
	Mild: 1	Mild: 30	Mild: 19		<5%: 2		
		Moderate: 0	Moderate: 4				
		Marked: 1	Marked: 3				
Right colon, pathologist 2 (*n*=61)	None: 59	None: 27	None: 50	None: 60	None: 60	None: 60	None: 59
	Mild: 2	Mild: 32	Mild: 8	Mild: 1	<5%: 0	Discrete, locally: 1	Regenerating epithelium: 2
		Moderate: 2	Moderate: 3		5–50%: 1		
Left colon, pathologist 1 (*n*=61)	None: 61	None: 53	None: 50	None: 61	None: 60	None: 61	None: 61
		Mild: 8	Mild: 9		<5%: 1		
			Moderate: 2				
Left colon, pathologist 2 (*n*=61)	None: 61	None: 54	None: 54	None: 61	None: 61	None: 61	None: 61
		Mild: 7	Mild: 7				

Biopsies from ileum was not assessed in one case.

Changes were more frequent in the right side of the colon (Table 2). Mild architectural changes, discrete local crypt degeneration, and regenerating epithelium was reported in biopsies from a few patients, while this was not seen in biopsies from the left side. A mild chronic inflammatory infiltrate in the lamina propria was present in the right side of approximately half of the patients, while this was a rare event in left-sided biopsies. In addition, an increased number of eosinophils were reported in a minority of patients as well as a few patients having biopsies with cryptitis in the right side of the colon.

### Lymphocyte counts

**Fig. 2:**
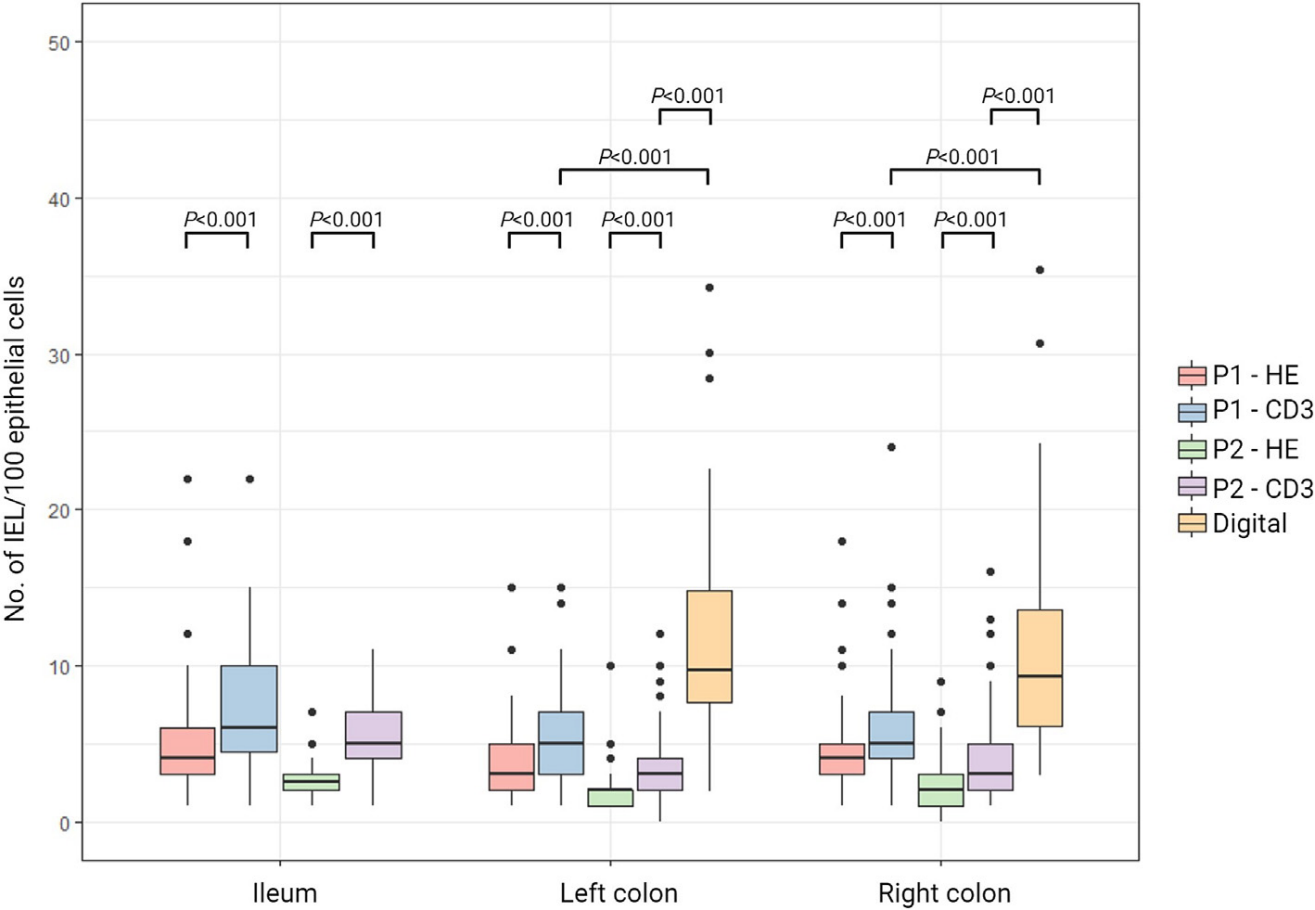
Comparison of counts of intraepithelial lymphocytes (IELs) assessed by Hematoxylin-and-Eosin (HE) and CD3 by each of the two pathologists and by digital evaluation according to intestinal localization. Dots represents outliers. IELs: Intraepithelial lymphocytes; P1: pathologist 1; P2: pathologist 2 (Two slides stained with HE and one slide stained with CD3 were not evaluated by pathologist 1 and one slide was nor assessed digitally.).

Fig. 2 visualizes the number of IELs in the surface epithelium according to manual and digital assessments, different stainings, and intestinal localization. Mean number of IELs by HE stain was 5.3 (95% CI 4.1–6.5, range 1–22) in the ileum, 4.6 (95% CI 3.8–5.3, range 1–18) in the right colon, and 4 (95% CI 3.3–4.7, range 1–15) in the left colon by one pathologist and 2.6 (95% CI 2.3–3, range 1–7) in the ileum, 2.4 (95% CI 2–2.8, range 0–9) in the right colon, and 2 (95% CI 1.7–2.4, range 1–10) in the left colon by the other pathologist. On CD3-stained slides, a significant higher number of IELs was reported by both pathologists in all localizations (*p*<0.001). Mean number of IELs by CD3 stain was 7.5 (95% CI 6.2–8.8, range 1–22) in the ileum, 6.3 (95% CI 5.4–7.2, range 1–24) in the right colon, and 5.3 (95% CI 4.6–6.1, range 1–15) in the left colon by one pathologist and 5.5 (95% CI 4.8–6.2, range 1–11) in the ileum, 4 (95% CI 3.1–4.8, range 1–16) in the right colon, and 3.5 (95% CI 2.9–4.2, range 0–12) in the left colon by the other pathologist. Thus, the registration of several both general histological parameters and number of IELs differed between the two pathologists.

**Fig. 3:**
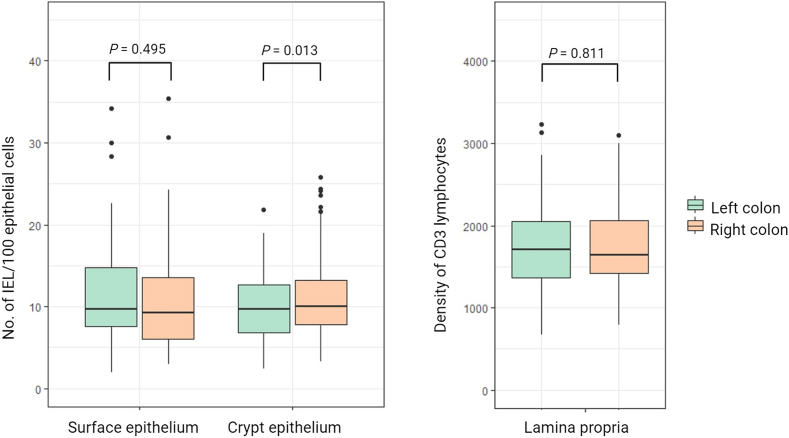
Comparison of digital counts of CD3-stained slides in surface epithelium and crypt epithelium and densities of CD3 positive cells in lamina propria in right and left colon. Dots represents outliers. (One case was not assessed).

Digitally obtained lymphocyte counts in the surface epithelium were significantly higher when compared with the manually obtained counts for both pathologists (*p*<0.001). The mean number of IELs in the right colon was 10.9 (95% CI 9.2–12.7, range 3–35) and 11.5 (95% CI 9.8–13.1, range 2–34) in the left colon. Digital lymphocyte counts were not only obtained in the surface epithelium, but also in the crypt epithelium and lamina propria. Fig. 3 depicts the number of lymphocytes both in the surface and crypt epithelium, as well as the density of CD3-positive lymphocytes in the lamina propria, according to the location in the colon. When comparing left and right colon, no significant differences were identified in the number of IELs in the surface epithelium, or in the density of CD3-positive lymphocytes in lamina propria. In crypt epithelium, a significant higher number of IELs was counted in the right side of the colon compared to the left side (*P*=0.013). However, when the analysis was performed without the outliers, the difference was no longer statically significant (data not shown).

### Subepithelial band

**Fig. 4:**
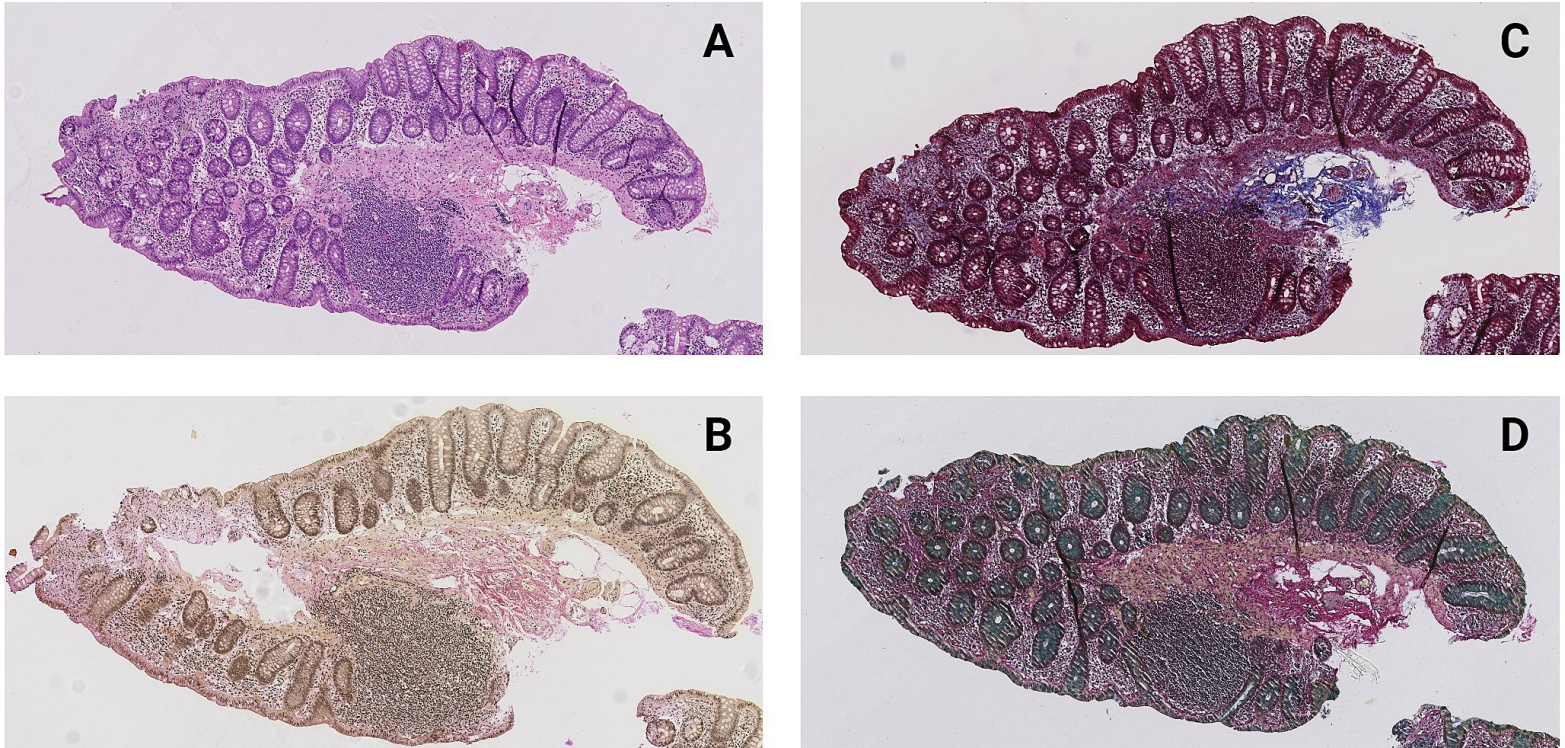
A section stained with (A) Hematoxylin-and-Eosin; (B) Van Gieson; (C) Masson Trichrome; (D) Weigert-Alcian-Sirius (magnification 7×).

In no cases, a convincing thickened collagenous band was present. However, Table 3 shows the thickness of a condensed subepithelial deposit according to intestinal localization. When the slides were assessed using a HE stain, a subepithelial deposit could be detected in 3.8% of the biopsies from the ileum, in 1.8% of the biopsies from the right colon, and in none of the biopsies from the left colon. When the thickness was assessed using special stainings (VG, MT, or WAS), a thin subepithelial deposit was visualized in every case, most frequently with a thickness of 1 μm. After adjustment for multiple comparisons, pairwise comparisons of the thickness according to different stainings showed significant difference when comparing the HE and each of the special stainings but not when comparing any of the special stainings (Table 4). Fig. 4 show an example of a biopsy with each of the four evaluated stainings.

**Table 3:** Thickness of collagen band (μm) according to different stains in the terminal ileum, right-, and left colon.

Thickness in μm	Hematoxylin-and-eosin	Van Gieson	Masson Trichrome	Weigert-Alcian-Sirius
*Ileum (n=52)*				
0, *n* (%)	50 (96.2)	0 (0)	0 (0)	0 (0)
1, *n* (%)	0 (0)	49 (94.2)	43 (82.7)	48 (92.3)
2, *n* (%)	2 (3.8)	2 (3.8)	8 (15.4)	4 (7.7)
3, *n* (%)	0 (0)	1 (1.9)	1 (1.9)	0 (0)
Not assessed, *n*	0	0	0	0

*Right colon (n=61)*				
0, *n* (%)	57 (98.3)	0 (0)	0 (0)	0 (0)
1, *n* (%)	0 (0)	57 (96.6)	51 (86.4)	56 (94.9)
2, *n* (%)	1 (1.8)	2 (3.4)	7 (11.9)	3 (5.1)
3, *n* (%)	0 (0)	0 (0)	1 (1.7)	0 (0)
Not assessed, *n*	3	2	2	2

*Left colon (n=61)*				
0, *n* (%)	59 (100)	0 (0)	0 (0)	0 (0)
1, *n* (%)	0 (0)	59 (96.7)	51 (83.6)	58 (95.1)
2, *n* (%)	0 (0)	2 (3.3)	10 (16.4)	3 (4.9)
3, *n* (%)	0 (0)	0 (0)	0 (0)	0 (0)
Not assessed, *n*	2	0	0	0

**Table 4:** *p*-Values when performing pairwise comparisons of the thickness of the collagenous band according to performed stainings in the ileum, right-, and left colon.

	Ileum	Right colon	Left colon
HE vs. WAS	<0.001	<0.001	<0.001
HE vs. MT	<0.001	<0.001	<0.001
HE vs. VG	<0.001	<0.001	<0.001
WAS vs. MT	0.031	0.109	0.039
WAS vs. VG	1	1	1
MT vs. VG	0.070	0.031	0.008

HE: Hematoxylin-and-Eosin; VG: Van Gieson; MT: Masson Trichrome; WAS: Weigert-Alcian-Sirius. After adjustment for multiple comparisons, a *p*-value <0.008 was considered statistically significant.

## Discussion

Being familiar with the spectrum of normal histology is fundamental in the daily diagnostic work of a pathologist. The normal range is not an exact science, and every day, estimates have to be performed by the individual pathologist. Usually, this is not problematic and has no clinical impact, but in some situations, only minor details discriminate normal from diseased tissue. New stainings and methods are continuously introduced, often with the purpose of increasing sensitivity. A more recent development in pathology is the introduction of digital image analysis and AI which have the potential to save time and create objective and highly standardized results.^29^ Careful evaluation and validation of new stainings and methods to establish their reliability, sensitivity, specificity, and clinical utility in normal tissue is important to avoid misinterpretation or overdiagnosis. The present study evaluates the number of IELs and presence of a subepithelial collagenous band in intestinal biopsies obtained from patients with absence of GI–symptoms (other than microscopic blood) and a macroscopic normal colon mucosa according to different stainings and imaging methods. This is in contrast to most previous studies examining the spectrum of normality that have included symptomatic patients with normal histology. Overall, higher numbers of IELs were reported when slides were assessed manually by CD3 compared with HE, and even more marked by digital evaluation. A thin subepithelial deposit was visualized with all connective tissue stains that could not be seen on HE.

General histopathological features were assessed based on the comprehensive scale that evaluates all components of the mucosa separately, described by Geboes and initially developed for assessment in patients with ulcerative colitis.^24^ A variety of unspecific changes were reported being most pronounced in the right-sided colonic biopsies. One or both pathologists reported mild changes comprising mildly elevated number of lymphocytes or eosinophils in the lamina propria in around half of the cases. As none of the patients had any GI complaints at the time of the study, nor a former or a later histological verified diagnosis of inflammation in the GI tract, this might reflect the wide range of normality. The pathologists were not asked to specify a diagnosis as part of the study and were aware that all biopsies originated from asymptomatic patients with a positive blood fecal test. Despite differences in specific parameters, the pathologists stated that, overall, all cases would have been signed-out all as normal or unspecific changes. Thus, the observed variability in some specific parameters between the two pathologists show that individual differences in histological evaluation was present, but with no significant influence on the final diagnosis. This is also supported by two previous studies establishing a high interobserver agreement discriminating MC from non-MC.^30^^,^^31^

The most recent European guideline concerning MC states that, in most cases, HE-stained tissue sections are sufficient although it is recommended always to use supplementary special or IHC-stainings in borderline cases.^10^ Several IHC markers highlighting different subpopulations of T-lymphocytes are used including CD3, CD8, and CD4.^32^, ^33^, ^34^ The choice of IHC stain is important, as the number of IELs is a bit higher on a CD3-stained slide compared with CD8-stained slide, while low IEL counts are reached with CD4.^32^^,^^33^ In the present study, the number of IELs was significantly higher when manually counted by CD3 compared with HE-stained slides in biopsies from all examined localizations. As it is unlikely that the number of IELs vary between HE- and CD3-stained slides, the study shows that identification and final counts of IELs depends on the stain. CD3 highlights the lymphocytes resulting in higher manual counts by the pathologist. The results are in line with previous studies examining biopsies from patients suspected for MC and coeliac disease.^35^^,^^36^ A concern has been raised of overdiagnosing LC/LCi if identical diagnostic criteria are applied using different stains.^13^^,^^35^ The results of the present study suggest that although <5 IELs is commonly observed in asymptomatic adults, higher values cannot be considered abnormal, and when using CD3, even higher numbers lie within the normal range, with maximum values reached in the ileum. Furthermore, our results showed that manual evaluation of IELs resulted in different counts by the two pathologists on both HE- and CD3-stained slides probably reflecting different estimates of both the number of negative epithelial cells and the number of positive lymphocytes.

Digital image analysis is emerging in several aspects of diagnostic pathology and opens up for performing standardized and very accurate quantifications.^29^^,^^37^, ^38^, ^39^ However, knowledge of expected differences in the obtained results when applying this method is crucial. A pronounced difference was seen when comparing the manually obtained IEL counts with digital measurements in the surface epithelium. In comparison to manual counts, the digital analysis performed the counting in the total surface epithelium and not only in hotspots. Still, even higher number of IELs were identified by AI. This is in accordance with a previous study using digital image analysis for assessment of the number of IELs in patients with LC.^40^ The digital analysis had the advantage of being able to perform exact counts and the slides were visually controlled by assure correct classification of the nuclei’s. Attention should be paid to this discrepancy before considering the introduction of an automated workflow.

International guidelines recommend sending biopsies from the right and left side of the colon in separate containers as normal histology is described to differ in relation to cellularity in lamina propria with the right side being more cellular.^10^^,^^20^^,^^41^^,^^42^ In agreement with this, several studies concerning MC have shown that changes in biopsies from the right side of the colon are more pronounced.^43^^,^^44^ The digitally evaluated number of CD3 positive cells in surface epithelium, and lamina propria did not differ significantly between biopsies from the right and left colon in this cohort questioning the difference between the segments. However, one limitation of the present study is that only T-lymphocytes were counted. Although the lymphocytes in the surface epithelium are mainly T-lymphocytes, plasma cells belonging to the B cell line are one of the main constituents in the lamina propria.^8^^,^^21^ One study have reported plasma cells to be the most commonly encountered cell in normal colon mucosa.^45^ A potential gradient with decreased cellularity in lamina propria when going from right to left side could thus be missed when only measuring densities of T-lymphocytes and can probably explain the discrepancy. Future studies should focus not only on T-lymphocytes but also plasma cells and eosinophils and include a higher number of patients to establish reliable measurements. Another limitation is that all tissue not identified as surface or crypt epithelium was classified as lamina propria without consideration of inevitable inclusion of lamina muscularis mucosa or even small part of submucosa in some cases, which would increase the area and thereby decrease the density of CD3 positive lymphocytes. Even so, none of the mentioned limitations are expected to have any influence on the number of IELs.

Only 12 slides were included in the training set to obtain a satisfactory result of the segmentation algorithm. This number is quite low and we assume this might reflect the fact that the morphological difference between the four compartments; background, surface epithelium, cryptal epithelium, and lamina propria is high, combined with a low variation in the morphology in normal mucosa between individuals. The morphological variation between patients in diseased tissue is higher and therefore the number of training slides would also need to be higher in this situation. Generally speaking ready-to-use algorithms like the nuclei detection APP used in this study is desirable. However, it is our experience that even though this APP was trained over a broad spectrum of stainings and tissue types, individual adaption is necessary. We have used this APP as the basis in several other studies for counting T-lymphocytes in a variety of settings and adaption for each individual study was needed even though the stainings was performed in the same laboratory.^46^, ^47^, ^48^ Thus, staining performed in other laboratories, use of other scanners or software are likely to result in different counts. Therefore, if implemented in the clinic individual customization of algorithms must be expected and external quality assessment might be needed similar to the IHC quality assessment performed by, e.g., NordiQC to detect differences between laboratories and provide guidance on how to obtain comparable results.

We also investigated presence and thickness of a potential subepithelial band by four different stainings. In relation to CC, connective tissue stainings as, i.e., VG, MT, Sirius red,^49^, ^50^, ^51^ or IHC staining with Tenascin^52^ have proven to be of value as the collagenous band is visualized and highlighted. In this study, all special stains revealed a 1–2 μm thick subepithelial deposit, while on HE stain, a subepithelial deposit was only rarely seen. However, in normal ileum and colonic mucosa, the choice of stain seems to be unimportant as presence of a thin subepithelial deposit has no clinical relevance and probably merely represents the basement membrane.

## Conclusion

Knowledge of normal histology is fundamental and used by the pathologist, mostly unconsciously, every day. In this study, different stainings were examined in biopsies from patients with macroscopic normal colon mucosa and no clinical symptoms. CD3 stain increased the number of identified IELs compared with HE-stained slides. An AI-based algorithm was developed and by digital image analysis even higher values was obtained. Several connective tissue stainings identified a thin subepithelial deposits not visualized by the HE-stained slides. Conclusively, the range of normality varies according to the staining and method used, which is important to be aware of when discriminating normal biopsies from biopsies with minor changes. Hopefully, an APP with the ability to discriminate normal from diseased colonic mucosa will be developed. Such an APP could be used to perform a primary screening of biopsies from patients with macroscopic normal colonic mucosa and act as a support for the pathologist that should only verify the automatic evaluation, or in the final scenario, the algorithm could perform automatic sign-out of normal biopsies. This would be a huge step forward that could save considerable time for GI pathologists. In order to achieve this, a much larger dataset would be needed including cases with different aspects of diseased mucosa. Furthermore, for use in a clinical setting, an algorithm should optimally be able to perform counts based on HE-stained slides.

## Funding

The study was supported by 10.13039/100009007The Region Zealand Health Research Foundation (Application number R17A40B32). Sponsor had no influence on the study design; in the collection, analysis and interpretation of data; in the writing of the report; or in the decision to submit the article for publication.

## Author contributions

Conceptualization and Methodology: AF, PE, UE, SW, LM

Data curation and investigation: AF, PE, UE, TB, JR, SW, WC, AD, LM

Formal analysis and visualization: AF, DJ

Funding acquisition: PE, LM

Project administration: AF, LM

Resources and validation: AF, PE, TB, JR, SW, WC, AD, LM

Software: AF

Writing - original draft: AF

Writing - review & editing: All authors

## Declaration of competing interest

The authors declare the following financial interests/personal relationships which may be considered as potential competing interests:

Lars Kristian Munck reports financial support to the study was provided by The Region Zealand Health Research Foundation. If there are other authors, they declare that they have no known competing financial interests or personal relationships that could have appeared to influence the work reported in this paper.
